# “Vessels in the Storm”: Searching for Prognostic and Predictive Angiogenic Factors in Colorectal Cancer

**DOI:** 10.3390/ijms19010299

**Published:** 2018-01-19

**Authors:** Adriano Angelucci, Simona Delle Monache, Alessio Cortellini, Monica Di Padova, Corrado Ficorella

**Affiliations:** 1 Department of Biotechnological and Applied Clinical Sciences, University of L’Aquila, Via Vetoio, Coppito 2, 67100 L’Aquila, Italy; adriano.angelucci@univaq.it (A.A.); alessiocortellini@gmail.com (C.A.); monica.dipadova@univaq.it (D.P.M.); corrado.ficorella@univaq.it (F.C.); 2 Medical Oncology Unit, St. Salvatore Hospital, 67100 L’Aquila, Italy

**Keywords:** VEGF-A, angiopoietins, VEGF receptors, angiogenesis, bevacizumab

## Abstract

High expectations are placed upon anti-angiogenic compounds for metastatic colorectal cancer (mCRC), the first malignancy for which such type of treatment has been approved. Indeed, clinical trials have confirmed that targeting the formation of new vessels can improve in many cases clinical outcomes of mCRC patients. However, current anti-angiogenic drugs are far from obtaining the desirable or expected curative results. Many are the factors probably involved in such disappointing results, but particular attention is currently focused on the validation of biomarkers able to improve the direction of treatment protocols. Because clinical studies have clearly demonstrated that serum or tissue concentration of some angiogenic factors is associated with the evolution of the disease of mCRC patients, they are currently explored as potential biomarkers of prognosis and of tumor response to therapy. However, the complex biology underlying CRC -induced angiogenesis is a hurdle in finding rapid solutions. The aim of this review was to explore molecular mechanisms that determine the formation of tumor-associated vessels during CRC progression, and to discuss the potential role of angiogenic factors as diagnostic, prognostic and predictive biomarkers in CRC.

## 1. Angiogenesis in Colon Cancer

**Figure 1:**
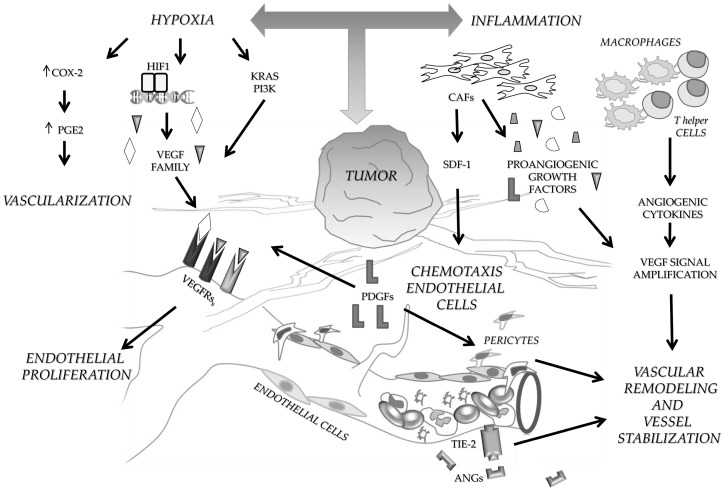
Schematic representation of the main molecular factors and events involved in angiogenesis during CRC progression. Hypoxia and inflammation are indicated as main driver events that through the interplay between tumor and normal associated cells enrich the tumor microenvironment with angiogenic growth factors. Abbreviations: cyclooxygenase (COX-2); prostaglandin-E2 (PGE2); GTPase KRas (Ki-Ras); phosphatidylinositol 3-kinase (PI3K); hypoxia-inducible factor 1 (HIF) 1; vascular endothelial growth factor receptors (VEGFRs); platelet derived growth factors (PDGFs); cancer associated fibroblasts (CAFs); stromal cell-derived factor 1 (SDF-1); angiopoietins (ANGs); tyrosine-protein kinase receptor TIE-2 (TIE-2).

Angiogenesis is a fundamental determinant of solid tumor progression and a promising target in cancer therapy. The formation of new blood vessels is realized through a stereotypical process involving a cascade of sequential steps that could be recapitulated during tumor growth. The pathways underlying cancer-stimulated angiogenesis are activated by adaptive or aberrant processes: in the first case the lowering oxygen availability in growing tumor mass can physiologically activate the hypoxia-associated adaptation program in cancer or in neighbor normal cells; otherwise, the angiogenesis can be aberrantly induced by cancer cells due to oncogenic transformation of key signaling pathways (Figure 1). In colorectal cancer, angiogenic switch occurs early in neoplastic progression in adenomas, and microvessel density correlates positively with formation of metastases and peritoneal dissemination [1,2]. Vessel count results are higher at the invasive edge of tumor than within the tumor [3]. Several angiogenic growth factors have been identified as expressed at high levels with vascular endothelial growth factor (VEGF)-A, which represents the most consistently expressed factors during CRC progression and metastatization. For other angiogenic growth factors, the panorama is more complex, suggesting for each ligand a role in a different time during normal–adenoma–carcinoma sequence [4].

### 1.1. Angiogenic Growth Factors 

Hypoxia is a frequent feature of solid tumors and its central role in activating angiogenic switch is increasingly highlighted. The continuous and dysplastic growth of tumor determines the formation of hypoxic microenvironment that induces the stabilization of HIF1-α and its association with HIF1-β). The heterodimer HIF1 determines adaptive phenotype in cancer cells exerting direct transcriptional activation of target genes. CRC is one of the cancers in which HIF1-α subunit is overexpressed from the early stages of carcinogenesis [5]. The presence of hypoxic zones in CRC aids dissemination of cancer cells and drug resistance [6]. Low oxygen tension, by increasing HIF1-α stability and dimerization, stimulates the transcription of several genes, including the VEGF-A. VEGF-A, a member of the VEGF family, and its receptor vascular endothelial growth factor receptor 2 (VEGFR)-2 are considered to be the leading determinants of angiogenesis. However, it was recognized that high HIF1-α expression is an independent prognostic marker for CRC regardless of VEGF upregulation [7]. Indeed, hypoxia-stabilized HIF1-α stimulates cyclooxygenase-2 (COX-2) mRNA expression, which in turn leads to enhanced prostaglandin E2 levels that are associated with increased CRC vascularization. For this reason, prostaglandin E2 was proposed as prognostic marker in CRC disease [8,9]. In agreement with this hypothesis, it was demonstrated that COX-2 inhibitors were able to reduce tumor-associated angiogenesis [10]. 

#### 1.1.1. VEGF-A

Several studies, both in vitro and in vivo, have demonstrated that VEGF-A is the key molecule in promoting angiogenesis and it is actively involved in tumor growth and metastasis. As is well known, VEGFs specifically bind to VEGF tyrosine kinase receptors on endothelial cells, including VEGFR-1 (alias Fms-like tyrosine kinase 1 (FLT1)) and VEGFR-2 (alias kinase insert domain receptor (KDR)), and they initiate intracellular signal transduction pathways associated with vascularization and angiogenesis in malignant tissue [11]. VEGFR-2 can transduce the full range of VEGFs responses in endothelial cells, controlling different steps during the formation of the vascular tube. The binding of VEGF-A with VEGFR-2 represents the most effective inducer of endothelial cell migration and proliferation and for this reason, the VEGF-A/VEGFR-2 axis is the gold target in current anti-angiogenic therapy for cancer. VEGF-A was found overexpressed in both tumor biopsy and in serum of CRC patients and it seems to be closely related to the severity of CRC and its clinical outcome [12,13,14]. Up-regulated VEGFR-2 has been detected, as expected, on tumor vessels supporting CRC, and, in a variable percentage of cases, also on CRC cells, suggesting a direct role in cancer cell biology apart from being a vasculature-restricted receptor [3]. Several studies have contributed to unveil a complex pattern associated with VEGF-A expression. Two families of VEGF-A isoforms are generated by alternate splice-site selection in terminal exon. The proximal splice-site selection results in at least six proangiogenic isoforms, and, of them, primary human CRCs can express four distinct variants: VEGF121, VEGF145, VEGF165 and VEGF189 [15]; VEGFA165 is the most biologically active isoform and it is secreted by most tissues. Although it is not completely elucidated, VEGF isoforms could exert different roles in modulating angiogenesis as suggested by their distinct binding capacities. VEGF121 isoform does not bind heparansulfate proteoglycan in the extracellular matrix, inducing a signaling response in endothelial cells that is, temporally and spatially, different compared with other isoforms [16]. Although missense point mutations appear uncommon in *VEGF* genes from cancer specimens, the natural high polymorphism of *VEGF* could provide an unexplored genomic basis for the diversity of angiogenesis programs among different patients [17]. Several single nucleotide polymorphism (SNP) haplotypes were identified in CRC, but few of them were common. In particular, the common −2578A, −460C, +405G and −2578A, −460T, +405G haplotypes were associated with the increased risk of CRC [18]. However, given the current literature, it is not possible to conclude that SNP in *VEGF* gene represent a potential risk factor for the development of CRC, as for other tumors.

#### 1.1.2. PlGF

Placenta growth factor (PlGF) is a member of the VEGF family exhibiting a strong angiogenic effect. The dimeric glycoprotein PlGF is a pleiotropic growth factor that works in synergy with VEGF-A, amplifying the downstream signal of VEGFR-1 and VEGFR-2 [19,20]. PlGF is expressed in human CRC tissue, and it correlates with micro-vessel density and cancer progression [21]. In preclinical models, hypoxia was able to induce PlGF in human CRC cell lines and in vivo also in endothelial cells [22]. PlGF represents a suitable therapeutic target because it is highly expressed on both endothelial and vessel-associated cells in many tumors, but it is scarcely detectable in healthy tissues [23,24]. 

#### 1.1.3. ANGs

Together with VEGF, ANGs are among the most important growth factors in regulating tissue repair and vascular homeostasis. Angiopoietin system is deregulated in many cancers and many different types of cancer cells can be directly responsive to these factors. The human ANG family includes the ANG-1, -2 and -4 secretory factors that are ligands of the transmembrane TIE-2, mainly expressed in endothelial cells. Although the precise regulatory role of ANGs remains controversial, studies with knockdown embryos have clearly demonstrated their involvement in the development of both vascular and lymphatic systems [25]. The best studied ANG-1 and ANG-2 factors seem to have complementary role in angiogenesis: while ANG1 helps to stabilize mature vessels, ANG-2 is associated with vascular remodeling [26]. In CRC patients, ANG-2 is expressed ubiquitously in tumor tissue, whereas ANG-1 expression is rare [27]. These data are compatible with a hypothesis in which blood vessel destabilization, operated by ANG-1, is a key step in CRC angiogenesis; however, it is commonly accepted that ANGs have a limited potency alone and that they should play in concert with other pro-angiogenic factors. 

#### 1.1.4. FGFs

Distinct classes of growth factors have been associated with the proangiogenic phenotype in CRC. The fibroblast growth factors (FGFs) are small heparin-binding growth factors comprising 23 members that bind to one or more of the four high-affinity fibroblast growth factor receptors (FGFRs). Although the precise function of FGFs in new vessels formation remains elusive, experimental data are coherent in stating that several members of this family are able to promote a strong angiogenic response. It is now accepted that the FGF system plays a critical role in several biological processes involving a variety of cell types including endothelial cells. The basic FGF (bFGF), a member of FGFs family, stimulates VEGF-A expression in endothelial and stromal cells, proposing a model in which the connective tissue contributes to the progression of vessel formation by a positive paracrine loop [28]. bFGF regulates the angiogenesis process through a dual mechanism: it stimulates VEGF-A expression in endothelial cells and stromal cells and it modulates VEGFR-2 signaling [29]. FGF signaling inhibition could affect angiogenesis due to VEGF-A insufficiency, but bFGF alone seems to induce only a modest maturation of new vessels. Carcinoma cells can express FGFRs and they may mimic stromal cells as main source of FGFs. In fact, several reports exist about FGFs and of FGFRs 1–4 being expressed in CRC, and there is some evidence regarding the role of the FGF signaling axis in sustaining the autonomous growth and invasion of cancer cell [30]. Some splice variants, such as FGFR1-IIc, have been detected in CRC but not in adenoma-derived cell lines [31]. FGF axis is able to sustain in an autocrine manner the release of VEGF-A in CRC cells, and this was demonstrated for fibroblast growth factor-7 (FGF-7) after binding with FGFR2 IIIb [32].

#### 1.1.5. PDGFs

An important family of growth factors accompanying angiogenesis is represented by PDGFs. The PDGF/platelet-derived growth factor receptor (PDGFR) system is physiologically activated during embryonic vasculogenesis and wound healing in adults. Generally, CRC is associated with overexpression of PDGFs and PDGFRs in tumor cells and/or in tumor-associated cells [33]. PDGFs in their monomeric form include four different members (PDGF-A/B/C/D), that become active in some of their possible dimerization forms (PDGF-AA/BB/CC/DD/AB). The three different dimeric forms of PDGFR (αα, ββ, αβ) could bind different PDGFs, but the downstream signaling and the biological effects are largely overlapped. PDGF-BB is the best characterized member in CRC progression and it is upregulated in blood and tissues from CRC patients [34]. It is known that PDGF-BB stimulates angiogenesis via three mechanisms: direct stimulation of endothelial cells proliferation; upregulation of VEGF-A, FGFs and erythropoietin in pericytes; and recruitment of endothelial precursor cells from circulation [35]. PDGF-BB has been shown to be associated with CRC stage and with increasing pericytes within tumors [36,37]. PDGF-BB produced by CRC cells recruits pericytes by providing the needed support for the formation of mature vessels and sustaining endothelial cell survival. In addition, also PDGF-BB and PDGF-AB were described as upregulated in CRC but their role in CRC-associated angiogenesis is unclear. 

### 1.2. Tumor Microenvironment and Angiogenesis

Local inflammation or the production of chemotactic factors by cancer cells results in the accumulation of a variety of non-tumoral cells that can promote tumor growth. CRC, similar to most other solid tumors, is infiltrated by immune cells, including tumor-associated macrophages (TAMs), T cells and dendritic cells, by myeloid-derived suppressor cells (MDSCs) and by CAFs [38]. All these cells have been involved in CRC-associated angiogenesis, although with different mechanisms. Some infiltrating cells are naturally equipped to release pro-angiogenic factors due to their physiological involvement in inflammation and wound repair. The importance of pro-inflammatory microenvironment in CRC progression was also demonstrated by a significant association between tissue accumulation of tryptase-positive mast cells, an increase of serum tryptase and microvascular density [39,40]. However, in many cases, the pattern of growth factors, released by cancer cells, is crucial in determining in a paracrine manner the pro-angiogenic phenotype of infiltrating cells. 

#### 1.2.1. TAMs

Macrophages infiltrating the tumor are represented by both classically activated macrophages (M1) and alternatively activated macrophages (M2), and the latter share tumor promoting activities, including angiogenesis [41]. Both M1 and M2 macrophages were observed in CRC but the M2 phenotype is frequently more represented [42]. M2 macrophages are known to accelerate tissue repair by releasing VEGF-A and matrix degrading enzymes: this function is particularly active in hypoxia. Macrophages can be visualized in the stroma of adenomatous polyps and CRC and in particular along the tumor front. A significant correlation was found between the number of infiltrating macrophages and microvessel density [43]. The importance of macrophages in CRC progression was confirmed by a syngeneic mouse cancer model in which macrophages promoted vascular tumor density and metastasis [44].

#### 1.2.2. CAFs

Fibroblasts are an important mesenchymal component in many tumors and their phenotypic characterization has evidenced similarities with myofibroblasts, a specialized cell type that plays a critical role during normal tissue wound repair. Stromal myofibroblasts participate in angiogenesis by providing a repertoire of secreted pro-angiogenic growth factors, including VEGF-A, bFGF, transforming growth factor-β 1 (TGF-β1) and PDGFs. In addition, myofibroblasts may elicit vasculogenesis by secretion of SDF-1, that is a potent chemotactic factor for endothelial cells [45]. CRCs with abundant myofibroblast-like CAFs are associated with shorter disease-free survival [46]. Myofibroblasts express an array of proinflammatory cytokines and chemokines, contributing to recruit immune cells to the local microenvironment. The chemokine, CC motif, Ligand 2 (CCL2), (alias monocyte chemotactic protein 1 (MCP1)), a member of the C-C chemokine family, is secreted by CRC-associated myofibroblasts and stimulates the recruitment of monocyte, macrophages and MDSCs [47,48].

#### 1.2.3. MDSCs and Lymphocytes

The number of infiltrating MDSCs correlates with the stage and metastatic burden [49]. MDSCs are key players in immunoediting and contribute to the unbalanced response of T helper cell subsets Th1/Th2/Th17 observed in CRC, with a prevalent production of regulatory tumor-promoting cytokines. In particular, Th2 and Th17 cells can promote tumorigenesis and/or progression by stimulating angiogenesis. Key factors in this process are Interleukin (IL)-6 and IL-17. IL-6 is particularly abundant in microenvironment with a prevalence of Th2 subset [50]. IL-17 is a well-established angiogenic cytokine that stimulates migration and cord formation of endothelial cells in vitro and the formation of new blood vessel in vivo [51]. Overall, several suggestions about the role of tumor-infiltrating cells propose that a potential new anti-angiogenic strategy could consider also the reprogramming of the tumor microenvironment.

### 1.3. Angiogenesis Induced by Oncogenic Signaling

The oncogenic transformation determining the autonomous upregulation of endothelial growth factors within tumor mass can sustain CRC angiogenesis. Because only a minor subgroup of highly aggressive CRCs harbors copy number amplification of *VEGFA*, the proangiogenic phenotype of CRCs should be based upon the deregulation of specific signaling pathways [52]. Molecular pathogenesis of CRC represents a prototypal model of carcinogenesis and cancer progression and it is sustained by aberrant modulation of few signaling pathways: WNT–β–catenin signaling pathway, the TGF-β1 signaling pathway, the epidermal growth factor receptor (EGFR)–mitogen activated protein kinase (MAPK) pathway, and the PI3K pathway. In many cases these pathways participate also in maintaining a pro-angiogenic phenotype both in hypoxia and normoxia. For example, KRAS and PI3K signaling enhances the hypoxic induction of VEGF-A, even when HIF1-α was silenced [53]. These effects could be exerted through the activation of pathways directed to alternative transcription factors, such as SP1 [54].

#### 1.3.1. TGF-β1

The TGF-β1 is a ubiquitous peptide with a prominent role in the inhibition of epithelial cell growth. However, as observed in many other carcinomas, CRC carcinogenesis is accompanied by the acquisition of resistance to the growth-inhibitory effects mediated by TGF-β1 [55]. TGF-β1 is a pleiotropic growth factor that, through binding to its receptor, endoglin exerts a central role in cancer biology. TGF-β1 can act on endothelial cells, inducing VEGF-A expression [56] and thus participating in realization of some steps of the angiogenesis process [57]. Up to 85% of CRC cell lines are resistant to TGF-β1 growth-inhibitory effects [58]. In addition, TGF-β Receptor type II (TGFR-2) and SMAD family member 4 (Smad4)—a downstream mediator of TGF-β1 signaling—resulted genetically inactivated in about 20% of human CRCs [59]. The resulting hypothesis suggests that the TGF-β1 secreted by CRC cancer cells is mainly involved in modifying the normal environment surrounding the tumor by promoting cancer progression. In fact, analysis of CRC tissue revealed the presence of high levels of TGF-β1 mRNA and protein, mainly in the advanced stages of progression [60]. In addition, although the TGF receptors are poorly expressed in CRC cells, contributing to resistance to TGF-β1 in tumor cells, endothelial cells surrounding cancer cells synthesize and express TGFb receptors [61]. The overexpression of TFG-β1 in CRC tissue leads to high serum levels in patients, and correlates well with microvascular density. Genetic studies have revealed a key function for TGF-β1 in vascular development and vascular homeostasis. *TGFB1* mutant embryos exhibit fragile vessels and in turn midgestation lethality, a phenotype exhibited also in mice with null mutations of other genes in TGF-β1 signaling pathways, including Activin receptor-Like Kinase (ALK)-1, (ALK)-5, endoglin and SMAD family member 5 (Smad5) [62]. TGF β stimulates the differentiation of precursors into pericytes and smooth muscle cells, and the deposition of extracellular matrix, allowing the stabilization of the vasculature. The stage of the progression and the microenvironmental conditions deeply influence the pro-angiogenic capacity of TGF-β1, generating a complex and unsolved scenario. Indeed, TGF-β1 seems to have an opposite effect on endothelial cells compared with VEGF-A, inducing antiproliferative effects and down-modulating VEGFR-2 mRNA and protein in a dose-dependent manner [63].

#### 1.3.2. WNT

The WNT/β-catenin pathway may also contribute to VEGF-A production and angiogenesis in CRC [64]. WNT signaling is important for tissue development and maintenance, but the aberrant activation of WNT signaling was frequently observed in CRC as a driver event. The oncogenic action of *WNT* on cell proliferation, migration and invasion is accomplished through the constitutive activation of β-catenin and, in turn, of lymphoid enhancer/T-cell factor (LEF/TCF) transcription factors [65]. Β-catenin signaling upregulates VEGF-A expression in vitro by direct binding of β-catenin/TCF4 to consensus binding sites within the gene promoter of *VEGFA* [66]. Another potential link between WNT signaling and angiogenesis involves the upregulation of pyruvate dehydrogenase kinase 1 (PDK1) that inhibits mitochondrial respiration by supporting glycolysis [67]. Because WNT signaling interferes with VHL expression, a negative regulator of HIF1-α, it was suggested that the observed WNT activation in CRC could be also contribute to the pro-angiogenic HIF1-α dependent phenotype [68]. 

#### 1.3.3. KRAS

About 40% of patients with CRC have constitutive activation of *KRAS*, and given the high concordance between primary tumor and metastases, the activation of this oncogenic step was considered fundamental in CRC progression [69]. KRAS is a membrane-anchored guanosine triphosphate/guanosine diphosphate (GTP/GDP)-binding protein involved in intracellular signal transduction downstream of protein kinase receptors, mainly epidermal growth factor receptor. It serves as a signal switch molecule that controls multiple cellular responses coupling receptor activation by specific growth factors with downstream effector pathways including the RAF–MEK–ERK and PI3K–Akt cascades. Several point mutations have been described for *KRAS*, approximately 80% occurring in codon 12 [70]. Although the oncogenic role of *KRAS* is well known, its prognostic role in CRC remains controversial [71]. The oncogenic activation of *KRAS* was associated with enhanced production of angiogenic factors including the chemokines, CXC motif, and VEGF-A [72]. Signaling through the KRAS up-regulates VEGF-A in a PI3K-dependent manner and this mechanism enhances pro-angiogenic stimulation by WNT signaling. Moreover, it was proposed that hypoxia itself could activate KRAS signaling also in absence of a mutated *KRAS* gene [73]. Constitutive KRAS signaling could also be transmitted by aberrant activation of RAF kinases, serine/threonine protein kinases that function as key downstream effectors of RAS. The RAF kinase family consists of three members: A-Raf proto-oncogene, serine/threonine (ARAF), B-Raf proto-oncogene, serine/threonine (BRAF), and Raf-1 proto-oncogene, serine/threonine (RAF1). Mutated *BRAF* gene was described mainly in melanoma patients, but about 10% of CRC patients are characterized by a valine-to-glutamate change at the residue 600 (V600E) of the protein. Although *BRAF* oncogene has attracted interest as a potential prognostic marker in CRC, its role in cancer progression is a subject of intense debate [74].

## 2. Targeting Angiogenesis in CRC: Current Clinical Outlook

The number of anti-angiogenic compounds that have been tested in clinical trials for metastatic (mCRC) patient treatment continues to grow (Table 1). They include both antibodies and small molecules with a great heterogeneity in their targets.

### 2.1. Bevacizumab

The first anti-angiogenic compound successfully used in metastatic mCRC patients was bevacizumab (Avastin^®^, Roche, Welwyn Garden City, UK), a recombinant humanized monoclonal antibody that inhibits VEGF-A. Bevacizumab is a favorable partner for combination chemotherapy; in fact, it does not generate cumulative toxicity, even if it is associated with specific side effects, such as hypertension, alteration of coagulation and thromboembolic phenomena. Several clinical trials have demonstrated that combining bevacizumab with a chemotherapy backbone improves clinical outcomes regardless of treatment protocol. Adding bevacizumab to treatment schedules containing a fluoropyrimidine (5fluorouracil or capecitabine) in combination with either irinotecan plus leucovorin (FOLFIRI) [86,87,88], or oxaliplatin plus leucovorin (FOLFOX) [89,90,91], has allowed to reach an overall response rate (ORR) higher than 50%, median progression free survival (PFS) between 8.3 and 11.1 months, and median Overall Survival (OS) between 20.3 and 22.2 months. The favorable toxicity profile permitted to develop intensive regimens, combining bevacizumab with triplet chemotherapies of 5fluorouracil, irinotecan and oxaliplatin. A randomized, open-label, multicentric phase III trial compared, in K/NRAS and BRAF unselected patients, the combinations folinic acid, 5fluorouracil, oxaliplatin and irinotecan (FOLFOXIRI) plus bevacizumab with FOLFIRI plus bevacizumab, both followed by a maintenance with bevacizumab and 5FU [92]. The study reached the primary end-point whit a median PFS of 12.1 months in the experimental arm (vs. 9.7 months, *p* = 0.006); ORR and median OS were respectively 65% and 31.0 months. Interestingly, maintaining VEGF inhibition with bevacizumab, beyond progression of disease, represents a valid therapeutic option in mCRC patients [93,94].

### 2.2. Aflibercept

More recently, aflibercept (Zaltrap^®^; Sanofi-Aventis, Frankfurt am Main, Germany)—another infused anti-angiogenic compound—has been introduced in clinical practice for second line treatment of metastatic CRC patients, following an oxaliplatin-based regimen. Aflibercept is a recombinant fusion protein consisting of VEGF-binding portions of the extracellular domains of human VEGF receptors 1 and 2 (VEGFR-1/VEGFR-2), fused to the Fc portion of the human IgG1 immunoglobulin. It binds to the circulating VEGF and it acts as a “VEGF trap”. Aflibercept inhibits the activity of VEGF-A and VEGF-B, as well as of PlGF. In a phase III study, considering K/NRAS and BRAF unselected patients, FOLFIRI plus aflibercept showed a statistically significant advantage in ORR, median PFS and median OS respect to FOLFIRI plus placebo. These positive clinical effects were confirmed also in patients previously treated with bevacizumab [95].

### 2.3. Vanucizumab

Vanucizumab is an anti-angiogenic bi-specific monoclonal antibody targeting VEGF-A and ANG2. In a recent double-blind, randomized phase II trial, which compared vanucizumab plus FOLFOX with bevacizumab plus FOLFOX as first line treatment of mCRC patients, the experimental arm failed to reach primary endpoint (median PFS improvement), and an alarming increase of blood pressure was observed in vanucizumab-treated cohort compared to control arm [83].

### 2.4. Regorafenib

Regorafenib (Stivarga^®^; Bayer Pharma, Leverkusen, Germany) is an oral multi-kinase inhibitor that acts on tyrosine kinases receptors (KIT, RET, PDGFR, FGFR) and serine/threonine kinases (BRAF). Its anti-angiogenic activity depends on blocking signaling downstream of VEGFR-2 and TIE2. After the phase III study which demonstrated a statistically significant benefit on OS vs. placebo [96], regorafenib became the standard therapeutic choice for metastatic CRC patients who are in disease progression after all standard chemotherapies.

### 2.5. TAS 102

Trifluridine/Tipiracil, also known as TAS 102 (Lonsurf^®^; AndersonBrecon Limited, Hereford, UK), is an oral mixture consisting of the cytotoxin trifluridine (TFT) and thymidine phosphorylase inhibitor tipiracil (TPI). In a phase III study, comparing TAS 102 with placebo, in metastatic CRC patients who had received at least two standard chemotherapic treatments, and had prior treatment with fluoropyrimidine, oxaliplatin, irinotecan, bevacizumab, and cetuximab or panitumumab, TAS 102 resulted in significant improvements of OS, median PFS and Disease Control Rate (DCR) [97]. Although TAS 102 has a prevalent genotoxic action, a preclinical model showed a potential role in angiogenesis exerted by inhibiting the angiogenic chemokine platelet-derived endothelial cell growth factor (PD-ECGF) [98]. 

### 2.6. Vandetanib

Vandetanib is an oral VEGFR-2 and Epidermal Growth Factor Receptor (EGFR) inhibitor currently tested in combination with cetuximab and irinotecan in a phase I study [81]. However, available data indicate for vandetanib an unsafe toxicity profile when combined with capecitabine, oxaliplatin and bevacizumab [82]. 

### 2.7. Nintedanib

Nintedanib is an oral VEGFR-1/-2/-3, FGFR-1/-2/-3 and PDGFR-α/β inhibitor. It showed, when combined with FOLFOX in a phase I/II study, comparable results respect bevacizumab as first line treatment in metastatic CRC patients [78]. Despite the absence of significant benefit compared to other anti-agiogenesis drugs, Nintedanib was tested in a phase III study plus the best supportive care in comparison with placebo plus the best supportive care [79]. 

### 2.8. Trebanabib

Trebanabib is an Fc-fusion protein that inhibits the interaction between ANG1/2 and the TIE2 receptor. Trebanabib failed to reach a statistically significant improvement in median PFS in a phase II, placebo-controlled, randomized study, in combination with FOLFIRI [80].

### 2.9. Vorinostat

Vorinostat is an histone deacetylase inhibitors, with epigenetic activity, and it has been shown to repress HIF1-α through translational inhibition [84], but a phase I/II study of vorinostat plus 5FU in mCRC pts with elevated intratumoral thymidylate synthase failed to determinate a maximum tolerated dose [85].

### 2.10. Fruquintinib

Fruquintinib is an oral VEGFR-1/-2/-3 inhibitor; in a phase Ib with subsequent randomized double-blind phase II study versus placebo, enrolling previously treated mCRC patients (≥than 2 lines), it showed a significant benefit in median PFS [99]. In the phase III confirmatory trial, fruquintinib showed statistically significant benefit in median OS, median PFS and response rate [77].

### 2.11. Famitinib

Famitinib is a wide spectrum kinase inhibitor targeting VEGFR-2/-3, PDGFRs, Stem Cell Factor receptor c-KIT, FLT3 and receptor tyrosine kinase RET. A multicentric randomized, double-blind, phase II study in mCRC patients in third or later line setting versus placebo showed for famitinib a statistically significant benefit in median PFS and disease control rate, without significant toxicity [76].

## 3. Angiogenesis-Related Prognostic Biomarkers in CRC

Angiogenesis plays a critical role in sustaining growth and progression of solid cancers, and for this reason angiogenic factors could represent useful prognostic biomarkers (Table 2). The greater the angiogenic potential of a tumor, the higher will be the proliferative rate and the invasive capacity. In addition, some angiogenic factors are measurable in serum or plasma, offering the possibility to perform a non-invasive and sequential screening. However, attention should be reserved to the modality of collection of blood specimens because it was proposed to influence significantly the detection of angiogenic biomarkers [100].

### 3.1. VEGF Signaling

VEGF-A is one of the most investigated biomarkers in CRC. The assessment of VEGF-A expression in tissues and/or in serum of cancer patients was used as an effective method in predicting metastasis from CRC [101,102,103]. Specifically, in a study conducted on seventy CRC patients, it was found that serum VEGF expression levels, determined by ELISA, were significantly correlated with advanced stage and metastases but not with age, gender, and tumor localization [14]. Another study, involving a cohort of one-hundred three CRC patients, reported that elevated circulating levels of VEGF were prognostic for liver and lung metastasis [104]. Kwon and colleagues have examined the expression levels of VEGF, IL-6 and C-Reactive Protein in patients who underwent curative resection for CRC and determined their reciprocal association and with histological findings. Also in this study, the VEGF level correlated significantly with tumor size and it has been proposed as a poor prognostic factor for overall survival [105]. Also, gene polymorphism in VEGF-A could hold prognostic information: a recent study demonstrated that the homozygous genotype VEGF-2578 AA had significant effect on time to tumor recurrence [106]. In order to strengthen the prognostic value of VEGF, some studies have suggested the parallel evaluation for circulating cytokines, including the chemokine, CXC motif, Ligand 1 (CXCL1), and IL-6 [104]. However, results from these studies were contradictory [105]. 

In order to circumvent the case in which angiogenesis does not determine a significant increase in circulating VEGF levels, biomarkers could be analyzed also in primary tumor site. The expression of VEGF was evaluated by immunohistochemistry in archived primary CRC tissue samples and it was found upregulated in combination with increased microvessel density (MVD), but authors failed to demonstrated a significant correlation with other prognostic factors and OS [107]. In association with VEGF-A expression, VEGF receptors VEGFR-1 and VEGFR-2 and/or their phosphorylation status have also been investigated. 

The detection of VEGFR-2 phosphorylated status, as reported by Giatramanolaki [108], in parallel with VEGF expression, could be a more reliable marker for active angiogenesis in tumors and may be useful in translational research with agents targeting VEGF-A. The phosphorylation status of pVEGFR-2 was determined on formalin-fixed paraffin embedded tissues demonstrating a significant difference in staining positivity between normal and cancer colon tissue. In particular, an elevated expression was detected in the cytoplasm and nucleus of cancer cells and in the tumor-associated vasculature, mainly at the invading tumor edge. This study also suggests that monoclonal antibodies raised against the phosphorylated form of VEGF receptors could be an useful prognostic/predictive tool in clinical management of CRC patients evidencing in a more specific manner the extension of the zones of active angiogenesis in the primary tissue [108]. Unfortunately, it has also been demonstrated that prolonged VEGFR-2 blockade, after an initial stabilization of the disease, leads to an upregulation of several other pro-angiogenic growth factors such as PDGFs, FGFs, ANGs and of tumor-angiogenesis related interleukins, thus lowering the prognostic value of VEGFRs [109]. 

### 3.2. ANG Signaling

Changes in the expression of ANGs and their receptors have been frequently reported in several malignancies and in mCRC. Available results are largely concordant in suggesting that Ang-2 over-expression is a frequent event in CRC progression and that Ang-2 and Tie-2 plasma concentrations are reliable prognostic markers. Indeed, numerous evidences demonstrated elevated serum and tissue levels of ANG-2 in CRC and the association between Ang-2 overexpression and lymph node metastasis, venous invasion and MVD [124]. Hong et al. in a retrospective study conducted by immunohistochemistry reported an increased expression of Ang-2 and a strong and inverse correlation with prognosis and OS. They concluded suggesting an important role of Ang/Tie2 signaling as additional tumor markers in CRC [125]. Another important study analyzed serum and tissue specimens from 490 patients with CRC to evaluate the significance of ANG-2 in both serum and primary CRC tissue [126]. In this study CRC patients showed the overexpression of ANG-2 in association with tumor progression. Similarly, another study conducted by Engin et al. demonstrated a significant increase in ANG-2 and Tie-2 plasma concentration in CRC patients whereas ANG-1 levels were not statistically different respect to control group. Moreover, ANG-2 concentration was correlated with the tumor stage [127]. Investigation about plasma ANG-1 expression has generated equivocal results. This could be because ANG-1 has an antagonistic role in regulating the angiogenesis process respect to ANG-2 role. Therefore, it has been proposed that a better estimation of prognosis would derive from the calculation of expression ratio ANG-2:ANG-1 [127].

### 3.3. PDGF Signaling

Manzat-Saplacan et al. investigated, by RT-PCR, in blood sample from CRC patients and control subjects, the expression of different genes involved in angiogenesis [128]. From the comparison emerged significant upregulation only for PDGF-C and clusterin. Even if other investigated factors showed an increased expression in CRC their difference respect to control group was not statistically significant. The prognostic role of PDGFs and PDGFRs has been investigated also in gastrointestinal cancers where PDGF-D and PDGF-C resulted associated with tumor progression recurrence, distant metastasis and poor outcomes [129,130,131]. However, despite several studies having analyzed the role of PDGFs as prognostic factor, the clinical and biological importance of PDGFs expression in human CRC is still debated [132].

### 3.4. FGF Signaling

bFGF, when upregulated, contributes in maintaining high levels of VEGF, and therefore it could be involved in resistance to anti-VEGF therapy. Initial evidence about the role of bFGF as prognostic factor in CRC was produced by Iwasaki and colleagues who analyzing resected tissue specimens from a small number of CRC patients, individuated a correlation between bFGF and tumor stage [133]. Another study, on 52 CRC patients, investigated the potential prognostic role of VEGF, bFGF and NO levels obtaining a significant increases only in VEGF and NO: the determination of serum levels of these factors was proposed to predict the progression of malignancy [134].

### 3.5. TGF-β Signaling

Because of its dual role as suppressor of carcinogenesis and promoter of cancer progression, expression levels of TGF-β1 have been studied mainly in patients with a late stage of CRC [135]. In particular, Chun and colleagues investigated, by immunohistochemistry, the expression levels of TGF-β1, TGF-β1 receptor, and downstream effectors in 201 cases of stage III rectal cancer: from the study emerged a correlation between low expression of TGF-β1 and poor prognosis [135].

### 3.6. Non-Coding RNA Signaling

Recently, because of their association with various cancers, including CRC, and their involvement in numerous biological processes such as angiogenesis, invasion and proliferation, non-codingRNAs (ncRNAs), mainly microRNAs (miRNA) and long non-codingRNA (lncRNAs) have gained growing attention. Since miRNA and lncRNAs seem to be stable in stool and easily measurable in blood plasma and serum, they can represent a new alternative and attractive strategy for developing prognostic and predictive biomarkers of CRC. It is well known that numerous microRNAs are able to regulate tumor angiogenesis, for example, by inhibiting components of the hypoxia signaling pathway and in this way blocking transcription of downstream elements that regulate angiogenic switch. This is the case of miR-22, that was found highly expressed in human CRC but it is absent in normal colon tissue. In fact, miR-22 inhibits HIF1-α expression, repressing VEGF-A production during hypoxia. Accordingly, knockdown of endogenous miR-22 enhanced hypoxia-induced expression of HIF1-α and VEGF-A [136]. Also, miR-499 regulates VEGF pathway under hypoxia–ischemia conditions and it seems to be involved in CRC angiogenesis by targeting WNT signaling [137]. Moreover, miR-135a/b and miR-17-92a cluster can activate the WNT signaling pathway via suppression of adenomatous polyposis protein (APC) or transcription factor E2F1, respectively [138,139]. A tumor-suppressing role in CRC has been proposed also for the miR-143 and miR-145. In particular, Chen and colleagues demonstrated that the inhibitory effect of miR-143 was dependent on the association between miR-143 and KRAS translation [140]. Later, other groups demonstrated a decrease of miR-143 and miR-145 expression in precancerous tissue and neoplastic colorectal tissue respect to normal mucosa. A possible link with angiogenesis was demonstrated for miR-145 that can target p70S6K1 downregulating the downstream expression of HIF1-α and VEGF-A [141,142]. 

Recent data suggest a prognostic role for lncRNAs, including H19, lincRNA-p21, Taurine upregulated 1 gene (*TUG1)* and Hox transcript Antisense RNA gene (*HOTAIR)*. All these molecules have been associated with increased angiogenesis in different tumor models and interestingly, they have been proven to be involved in CRC metastasis [143]. Therefore, it is plausible that lncRNAs could modulate CRC progression by partly regulating angiogenesis. However, further data are needed in order to confirm this hypothesis.

### 3.7. Other Signaling Pathways

HIF1-α expression, in association with VEGF, is related to clinical outcome and prognosis: Wu Yugang et al. found elevated expression levels of HIF1-α, the chemokine, CXC motif, Receptor 4 (CXCR4) and of VEGF-A were significantly correlated with tumor stage, and progression [144]. The same conclusion was proposed in a different study where HIF-1α expression was evaluated by quantitative PCR and immunocytochemistry [145].

Several studies have established a strong association between tumors and chronic inflammation, demonstrating the overexpression of several inflammatory cytokines, such as IL-1, IL-6, IL-4 or IL-8 in tumor tissue or serum of cancer patients [146,147]. It has been reported that some of these cytokines are involved in promoting growth and progression of CRC. So, the determination of inflammatory cytokines in serum of CRC patients could be a useful tool for prognosis and diagnosis [148]. Citokines can affect the proliferation and migration of endothelial cells and promote angiogenesis [146,149,150]. The presence of hypoxic zones could further exacerbate this manifestation, as described for the secretion of IL-8 [151]. Evaluation of circulating levels of IL-1, IL-6, IL-8, VEGF and other cytokines, in a cohort study of sixty-nine patients, showed a significant correlation between IL-8 and VEGF, with recurrence in CRC [152]. Also, IL-6 can play a central role in cancer invasion and spreading, and it has been reported that in the presence of increasing levels of IL-6, both the OS and PFS of CRC patients worsened [153]. Nastase and colleagues analyzed by ELISA and quantitative PCR method, IL-8 expression levels in 62 CRC patients, finding a significant correlation between serum IL-8 levels and tumor stage [149]. Another study evidenced the association between IL-8 expression and tumor promotion suggesting a prognostic role in CRC for this interleukin [121].

## 4. Response Biomarkers in Anti-Angiogenesis Therapy of CRC

A growing list of factors has been investigated in the last years in order to individuate biomarkers able to predict clinical response to anti-angiogenesis drugs (Table 2). Nevertheless, the complexity of the underlying signaling pathways represents an important obstacle in finding a single effective biomarker. Moreover, there is a series of collateral problems that to date complicated the identification and validation of biomarkers. For example, the definition of objective response criteria suitable for anti-angiogenic therapy or the lack of reproducibility in biomarker measurement. Other problems can derive from the type of protocols routinely used for cancer treatment usually based on the combination of anti-angiogenic agents with a backbone of chemotherapy. Unfortunately, to date no absolute conclusion has been achieved, and clinical practice is still suffering from a lack of biological tools to select CRC patients who may benefit more than others from anti-angiogenetic treatments and monitoring them during treatments, in order to anticipate diagnosis.

Although initial evidence suggested that a rapid increase in the serum VEGF-A concentration may be a potential predictor of resistance to bevacizumab, Alidzanovic et al. [111] demonstrated that the increase of VEGF circulating levels, during bevacizumab-based treatment, should not be considered as a tumor escape mechanism, but rather a pharmacodynamic effect: the measured VEGF is largely complicated by bevacizumab [110]. Hegde et al. measured serum VEGF-A levels by ELISA at baseline, in a large cohort of cancer patients (colorectal cancer, renal cancer and lung cancer) from four randomized phase III studies with experimental bevacizumab-containing regimens [113,114]. The results of this study confirmed that higher pre-treatment VEGF-A levels had a prognostic significance, but they were not predictive for response to bevacizumab treatment. A recent retrospective analysis of the phase III study, comparing FOLFIRI plus aflibercept versus FOLFIRI plus placebo as second line treatment in CRC patients [95], analyzed the clinical response of bevacizumab-pretreated subgroup, measuring nine potential biomarkers implicated in angiogenesis. In this study, a positive correlation between disease progression and increased PlGF and VEGF-A concentrations was found. The authors concluded that aflibercept would be an optimal choice in patients progressed to prior bevacizumab-based treatment showing a rise of PlGF, VEGF-A and other angiogenesis circulating biomarkers [123]. A potential use of soluble VGFR-1, an endogenous blocker of VEGF, was proposed as biomarker to stratify patients with localized rectal cancer. In fact, plasma pretreatment sVEGFR-1 concentration was associated with both primary tumor regression and the development of adverse events after neoadjuvant bevacizumab and chemoradiation [120]. The predictive role of VEGF was evaluated also analyzing mRNA expression in circulating cells. Marisi et al. observed that a reduction in VEGF expression in plasma samples of metastatic CRC patients, from baseline to the first clinical evaluation, was correlated with a better outcome to bevacizumab-based treatment [154]. 

The *VEGFA* SNPs have also been investigated for their potential involvement in patients’ response to bevacizumab. Formica et al. firstly found a statistically significant association between VEGF gene polymorphisms (GPs) −152 (G/G vs. G/A + A/A), −1154 (G/G vs. G/A + A/A) and PFS [116]. In agreement, Koutras et al. confirmed the increased frequency of VEGF GP −1154 G/G in patients not responding to bevacizumab treatment and with a poor PFS [118]. Loupakis et al. retrospectively analyzed VEGF SNPs in 111 consecutive metastatic CRC patients treated with FOLFIRI plus bevacizumab, in order to evaluate their correlation with PFS [115]. The VEGF GP −1498 T/T genotype was associated with shorter median PFS in bevacizumab-treated patients, but not in the control group treated with FOLFIRI alone. Their findings seem to suggest a possible role of VEGF −1498 C/T variant in predicting the efficacy of bevacizumab, but a subsequent prospective study did not validate this hypothesis [117]. Although data need to be further validated, the predictive role of polymorphisms in other genes involved in angiogenesis has been proposed. Di Salvatore et al. showed a statistically significant association between IL-8 GPs −251 T/A and A/A and shorter PFS and OS, compared to TT alleles in bevacizumab-treated RAS mutant, metastatic CRC patients [122].

Although based on preliminary data, and on the analysis of a limited number of patients, the hypothesis is emerging of a promising predictive role also for VEGF isoforms. Bates et al. demonstrated that the ratio of VEGF-165b:VEGF-total (VEGF-165b is the predominant anti-angiogenetic isoform), analyzed by immunohistochemistry staining of tumor tissues, significantly correlated with disease free survival in patients being treated with bevacizumab and oxaliplatin-based chemotherapy [155]. In addition, Bunni et al. showed that plasma VEGF-Axxxb levels correlated with tissue VEGF-Axxxb expression, suggesting that also circulating levels of VEGF isoforms could be useful as a predictive biomarker for responsiveness to bevacizumab [156]. 

Recent studies have suggested that VEGF receptors, which have been shown to be independent prognostic markers in CRC, can be also considered in order to improve the predictive value of VEGF-A during anti-VEGF treatment [119,157,158]. Indeed, a diagnostic approach able to describe the pattern of the activation of signaling pathways involved in angiogenesis should result in a more predictive capacity. In fact, many of the currently evaluated anti-angiogenesis drugs are tyrosine kinase inhibitors that target factors downstream of VEGF. In addition, the investigation of the active signaling pathways could permit to predict the development of evasive resistance mechanisms usually associated to anti-VEGF monotherapies [159].

Lieu et al. measured levels of PlGF, VEGF-A, VEGF-C and VEGF-D in sequential plasma samples of metastatic CRC patients treated with chemotherapy plus bevacizumab, and of control cohorts subjected to regimens with or without bevacizumab [160]. Patients who progressed on chemotherapy with bevacizumab had significantly higher levels of PlGF, but not of VEGF-C and VEGF-D compared to patients treated with chemotherapy alone. Martinetti et al. aimed to validate the prognostic role of circulating VEGFs, PDGFs, Stromal Cell-derived Factor-1 (SDF-1), osteopontin and Carcinoembryonic Antigen, by analyzing plasma samples of patients treated with three bevacizumab-based regimens at baseline and during therapy [112]. They found that baseline higher levels of CEA, SDF-1 and VEGF were associated with poor prognosis, even if the correlation did not reach statistical significance.

To date, few consistent reports about the predictive role of miRNAs in CRC angiogenesis have been provided. Boisen and colleagues conducted an early study showing that some miRNAs expressed in tumor tissue and potentially involved in angiogenesis pathway have a predictive role on tumor response in patients treated with bevacizumab [161]. In addition, Hansen investigated the predictive role of circulating miR-126 measured by qPCR in mCRC patients, before, during and at progression to first-line chemotherapy with bevacizumab [162]. These findings about a significant concordance between miR-126 levels and tumor progression confirmed a possible predictive role of miR-126 in bevacizumab-based treatments.

## 5. Conclusions

More than forty years have passed since the first intuition about angiogenesis as an effective target in cancer therapy, yet angiogenesis is still a largely enigmatic phenomenon, involving the coordinated action of many players. Preclinical experimentation is actively involved in testing several new inhibitors against both the gold target VEGF and new potential angiogenic targets. Nevertheless, in many cases, expectations have not been followed by effective results in clinical trials. More information has to be known about mechanisms allowing the correct coordination of sequential steps in the formation of new vessels, and about the redundant effect of numerous factors involved. This aspect is further complicated by the fact that in tumor we usually assist at the realization of an “imperfect”, non-canonical angiogenesis. The clinical need for predictive biomarkers in anti-angiogenesis therapy is prompting researcher for a gold rush that is revealing itself to be a fishing expedition, generating results that are frequently difficult to compare because of heterogeneity in the methodological approaches. Currently there is not an angiogenic biomarker that could be used in clinical practice, although some important points have been clearly delineated: VEGF-A is a solid prognostic biomarker, but its predictive role as a unique biomarker is inadequate; different factors should be considered in parallel with VEGFs, taking into account also factors related to maturation and stabilization of new vessels; microenvironment should not be considered simply a bystander in angiogenesis and tumor-associated normal cells are important contributors in this process; tumor-angiogenesis is a dynamic phenomenon and angiogenic biomarkers could change in the progression of the disease and in relation to therapeutic protocol, making their prognostic value inadequate 

## Figures and Tables

**Table 1:** Anti-angiogenesis drugs approved for metastatic CRC and recent clinical trials with potential new anti-angiogenesis therapeutics.

Compound	Category	Pro-Angiogenic Targets	Combination Drugs	Clinical Use	Route of Administration	Ref.
Aflibercept	”Trap”-inhibitor, recombinant fusion protein.	VEGF-A/B, PlGF	Fluoropyrimidines, folinic acid and irinotecan	Second line, after oxaliplatin; regardless *K/NRAS* and *BRAF* genotype	Intravenous	[75]
Bevacizumab	Recombinant humanized—mAb	VEGF-A	Fluoropyrimidines, folinic acid, irinotecan and/or oxaliplatin	First and second line; regardless *K/NRAS* and *BRAF* genotype	Intravenous	[75]
Famitinib	Small molecule, multiple kinase inhibitor	VEGRs, PDGFRs, c-KIT, FLT3, RET	Alone	No, experimental use	Oral	[76]
Fruquintinib	Small molecule, multiple kinase inhibitor	VEGRs	Alone	No, experimental use	Oral	[77]
Nintedanib	Small molecule, multiple kinase inhibitor	VEGFR 1-3, FGFRs, PDGFRs	5FU, folinic acid and oxaliplatin	No, experimental use	Oral	[78,79]
Regorafenib	Small molecule, multiple kinase inhibitor	KIT, RET, PDGFR, FGFR BRAF	Alone	After progression to other conventional treatments	Oral	[75]
Trebanabib	Fusion protein	ANG-1/2, TIE2	5FU, folinic acid and irinotecan	No, experimental use	Oral	[80]
Trifluridine/tipiracil	Antimetabolite	PD-ECGF	Alone	After progression to other conventional treatments	Oral	[75]
Vandetanib	Small molecule, multiple kinase inhibitor	VEGFR-2, EGFR	Irinotecan and cetuximab; capecitabine, oxaliplatin and bevacizumab	Not in use, due to unsafe toxicity profile	Oral	[81,82]
Vanucizumab	Bi-specific monoclonal antibody	VEGF-A, ANG-2	FOLFOX	No, experimental use	Intravenous	[83]
Vorinostat	Histone deacetylase inhibitors	HIF1-α	5FU	No, experimental use	Oral	[84,85]

Abbreviations: FOLFOX (fluorouracil, leucovorin and oxaliplatin); 5fluorouracil (5FU); Cell Factor receptor c-KIT (c-KIT); FMS-like tyrosine kinase-3 receptor (FLT3).

**Table 2:** Prognostic and predictive biomarkers proposed for advanced CRC and validated by clinical experimentation.

Biomarker	Drug/Treatment	Prognostic Value	Predictive Value	Ref.
Circulating VEGF-A	FOLFIRI plus bevacizumab	Yes	Yes (VEGF-A ↑ during treatment and poor prognosis)	[110]
	XELOX, FOLFOX6 or FOLFIRI/FOLFOXIRI plus bevacizumab	Yes	No	[111]
Tissue VEGF expression	Randomized, 3-arms, phase II trial plus bevacizumab; multiple treatments	Yes	N/V	[112,113]
	FOLFIRI plus bevacizumab	N/V	Yes (↓ peri-therapeutic VEGF-A expression predicts responsiveness to bevacizumab and PFS)	[94]
	IFL	NO	NO (bevacizumab improves survival regardless VEGF levels)	[114]
SNPs VEGF	FOLFIRI plus bevacizumab, retrospective analysis	N/V	Yes (VEGF GP −1498 T/T genotype was associated with shorter PFS)	[115,116]
	FOLFIRI plus bevacizumab	N/V	No	[117]
	FOLFIRI AND XELIRI plus Bevacizumab	N/V	Yes (the VEGF GP 1154 G/G is associated with OS)	[118]
	Retrospective analysis	Yes (VEGF-2578 is associated with time of recurrence)	N/V	[106]
	FOLFIRI plus bevacizumab	N/V	Yes (VEGF GPs −1154 G/G −152 G/G is predictive for PFS)	[116]
Tissue VEGFR1,VEGFR2, VEGFR3 expression	CBI or CB *	Yes (VEGFR2 could predict clinical outcome in mCRC	N/V	[119]
	Four cycles of therapy plus Bevacizumab	N/V	YES (Pretreatment ↑ soluble VEGFR-1 is associated with higher response to therapy)	[120]
Tissue IL-8 and SNP expression	Retrospective analysis **	Yes	N/V	[121]
	Bevacizumab-based first line treatment	N/V	YES (IL-8 GPs-51 T/A and A/A are associated with shorter PFS and OS)	[122]
Serum PLGF	FOLFIRI plus bevacizumab/aflibercept	N/V	YES (↑PLGF and VEGF correlate with response in patients previous treated with bevacizumab)	[123]

* Independently of KRAS mutation; ** Various therapy protocols not reported in the article. N/V: not verified; CBI: cetuximab, bevacizumab, irinotecan; FOLFIRI: fluorouracil, leucovorin, irinotecan; FOLFOX: fluorouracil, leucovorin and oxaliplatin; IFL: irinotecan, fluorouracil, leucovorin; XELOX: capecitabine, oxaliplatin.
